# Smokers’ and E-Cigarette Users’ Perceptions about E-Cigarette Warning Statements

**DOI:** 10.3390/ijerph13070655

**Published:** 2016-06-30

**Authors:** Olivia A. Wackowski, David Hammond, Richard J. O’Connor, Andrew A. Strasser, Cristine D. Delnevo

**Affiliations:** 1 Center for Tobacco Studies, Rutgers School of Public Health, New Brunswick, NJ 08901, USA; 2 School of Public Health and Health Systems, University of Waterloo, Waterloo, ON N2L 3G1, Canada; 3 Department of Health Behavior, Roswell Park Cancer Institute, Buffalo, NY 14263, USA; 4 Department of Psychiatry, Perelman School of Medicine, University of Pennsylvania, Philadelphia, PA 19104, USA

**Keywords:** e-cigarettes, warnings, risk communication, health communication, risk perceptions

## Abstract

Cigarette warning labels are important sources of risk information, but warning research for other tobacco products is limited. This study aimed to gauge perceptions about warnings that may be used for e-cigarettes. We conducted six small focus groups in late 2014/early 2015 with adult current e-cigarette users and cigarette-only smokers. Participants rated and discussed their perceptions of six e-cigarette warning statements, and warnings in two existing Vuse and MarkTen e-cigarette ads. Participants were open to e-cigarette warnings and provided the strongest reactions to statements warning that e-liquid/e-vapor or e-cigarettes can be poisonous, contain toxins, or are “not a safe alternative to smoking”. However, many also noted that these statements were exaggerated, potentially misleading, and could scare smokers away from reducing their harm by switching to e-cigarettes. Opinions on the Food and Drug Administration’s proposed nicotine addiction warning and warnings that e-cigarettes had not been approved for smoking cessation or had unknown health effects were mixed. Participants perceived MarkTen’s advertisement warning to be stronger and more noticeable than Vuse’s. Care should be taken in developing e-cigarette warnings given their relative recentness and potential for harm reduction compared to other tobacco products. Additional research, including with varied audiences, would be instructive.

## 1. Introduction

Warning labels on cigarette ads and packages have been a traditional, visible and cost-efficient source of risk information about cigarettes [1]. Cigarette warning labels have been associated with smokers’ increased knowledge about tobacco health risks, motivation to quit, use of cessation services and smoking abstinence [1,2]. Previous research and recommendations suggest that these warnings should be specific, direct, and prominent, use pictorial images and take up significant pack space in order to maximize their effectiveness [1,2,3,4,5]. Warnings with graphic or shocking imagery of health effects or human suffering that can stimulate negative emotions such as fear seem to be particularly effective [1]. However, research on warning labels for other tobacco products has been very limited to date, and the content and style of these may need to be unique given the potential differences in their health effects and use [1]. This may be particularly true for tobacco products which may pose lower risks to individual users than combustible cigarettes. Indeed while the 2009 Tobacco Control Act mandated that cigarette warnings be updated to graphic warnings covering the top 50 percent of the front and rear panels of cigarette packages, it did not similarly require pictorial warnings for smokeless tobacco products and increased its required text warning size to 30% rather than 50%. Another newer non-cigarette product for which warnings may be considered are electronic cigarettes or “e-cigarettes”.

In May 2016, the Food and Drug Administration (FDA) published a deeming rule extending its tobacco regulatory authority to e-cigarettes, a move which will require e-cigarette companies to register their products with the FDA, and which bans free samples, and creates a minimum age-of sale for e-cigarettes [6]. In addition, the FDA’s deeming rule requires that e-cigarettes begin carrying a warning that they contain nicotine, an addictive chemical [6]. Although this would be an important first step in formally warning the public about their potential risks [7], some have called for additional and stronger warnings [6,8] and some e-cigarette brands and products have voluntarily started carrying their own warning messages, although without standardization the content and display of these can vary considerably [9,10,11]. Meanwhile, the public may also be encountering risk messages and warnings about e-cigarettes from other sources such as the news, health professionals, online media and friends and family [12]. However, little to date is known about how consumers might interpret such warning messages and what potential effects they might have.

Since the FDA’s deeming rule was initially proposed in April 2014, a few experimental studies have begun addressing the topic of e-cigarette warnings. Sanders-Jackson et al. (2015) tested e-cigarette warning statements embedded in television e-cigarette advertisements that aligned with two message frames commonly used in anti-smoking campaigns (ingredient-themed and tobacco-industry themed) and found that exposure to ads with these was associated with reduced e-cigarette cravings (among those with any e-cigarette cravings to begin with) and purchase intentions among young adults [13]. Another study examined the impact of warning messages on non-tobacco users’ perceptions of moist snuff, snus and e-cigarettes based on current smokeless tobacco warnings (i.e., “this product is not a safe alternative” and “this product can cause mouth cancer”) and a reduced risk message tobacco companies have proposed for snus products [14]. Authors found that the “not safe alternative” and oral cancer warnings both increased perceptions of e-cigarettes’ harmfulness, while the reduced risk message did not significantly alter harm perceptions. A discrete choice experimental study in Canada found that health warnings were more influential than e-cigarette flavors, price and nicotine content on participants’ intention to try e-cigarettes and that participants either preferred e-cigarettes with no warning label or with the most comprehensive label presented [15]. Only one study thus far has examined effects of a warning label already in use by a major e-cigarette brand. In this study, participants exposed to a detailed voluntary warning label adopted by the e-cigarette brand MarkTen were more likely to agree that it contained dangerous chemicals and could be dangerous to health than those in a no warning label control condition [9]. In addition, 42% of participants who currently used both cigarettes and e-cigarettes indicated that the warning made them think about quitting use of e-cigarettes.

Together, these few studies suggest that e-cigarette warnings may impact e-cigarette risk perceptions and use intentions but more research is needed including on how consumers might interpret various warnings. Our study aims to contribute to this new research area by starting from a more ground up approach using qualitative research. As the first part of a multi-step formative research project about e-cigarette risk messages and warnings, we conducted a set of focus groups with those adults most likely to encounter e-cigarette warnings because of their current product use or potential future use—i.e., current smokers and current e-cigarette users. Inclusion of both of these groups is important because in making policies about tobacco products under its authority, FDA must consider the potential impact to both users and potential users of those products, including the likelihood that existing users may stop using the product and non-users may start using the product [6]. We intended to gauge smokers and e-cigarette users’ perceptions of a set of e-cigarette warning statements which smokers and e-cigarette users may already be encountering or may encounter in the future in order to explore their potential impact and to inform future research questions and approaches to studying this topic. Using ads from two leading e-cigarette brands carrying a voluntary warning statement, we also aimed to provide preliminary data about e-cigarette warning formatting display considerations to support policy on this topic.

## 2. Materials and Methods 

### 2.1. Participants

We conducted six small in-person focus groups—three with current e-cigarette users (i.e., smokers or former smokers who have used e-cigarettes for more than one day in the last 30 days) and three with current smokers (i.e., have ever smoked 100 cigarettes and now smoke every day or some days) who may have ever tried e-cigarettes but were not currently active users (referred to from here on as “non-e-cigarette users”). We aimed to keep the groups small, and conduct these as “mini-focus groups” [16,17,18] (with 3–6 participants each), given our intent for each participant to discuss their individual perceptions of multiple presented warning messages. Two groups had 3 participants, 3 groups had 5 and one group had 6 participants, for a total of 27 participants (13 non-e-cigarette users, 14 current e-cigarette users). Most participants were male (18/27), had at least some college or technical school education (20/27) and were white (17/27). The remaining participants were black (4), Asian (4), or of other racial/ethnic backgrounds (2). Participants ranged in age from 19 to 58 and e-cigarette users were younger on average (27 years) than the non-user participants (40 years). All of the non-e-cigarette users were current smokers and most (77%) were daily smokers who had made a quit attempt in the last month (69%), intended to quit in the next six months (77%) and had ever tried e-cigarettes before (85%). Among current e-cigarette users, 8/14 still smoked tobacco cigarettes every day or somedays. On average, these dual users used e-cigarettes on 21 of the last 30 days and most (5/8) had used e-cigarettes more than 50 times in their life. The rest (6) of the current e-cigarette users were former smokers who now exclusively used e-cigarettes. All of these participants had used e-cigarettes more than 50 times and used e-cigarettes an average of 29 of the past 30 days.

### 2.2. Recruitment

Participants were recruited from an ad posting in the central New Jersey section of the classified advertisements website Craigslist. E-cigarette users were also recruited from flyer postings in two local vape shops and subject referrals from another unrelated e-cigarette study. Groups were conducted between December 2014 and January 2015.

### 2.3. Procedures and Stimulus Materials

Focus groups were approximately 90 minutes each and began with questions about participants’ exposure to and experience with e-cigarettes, and perceptions about their potential risks and benefits. Next, participants viewed and provided feedback on six e-cigarette warning statements. Since the range of specific e-cigarette health risks is still unknown, we intended to gain feedback on basic factual warning points which might serve as logical starter warning statements for e-cigarette ads, products and related packing. The statements used were informed by preliminary recommendations for e-cigarette warnings made in the tobacco control community, points already included in some delete space here e-cigarette packaging or advertising, warnings commonly stated by health and public health professionals and the research team’s experience with cigarette warning label research [1,2,6,13,14,15]. The six tested concept statements are found in Table 1. For two of the messages, slight variations in wording were presented in the last two groups based on feedback from the previous sessions and one study that emerged during the time of data collection (see Table 1). The presented order of the six statements was randomized across each of the three non-e-cigarette user groups. Current e-cigarette user groups viewed the statements in the same randomized order as the non-e-cigarette user groups. During the sessions, the messages were presented to participants one at a time on an overhead projector and on individual pieces of paper passed to each participant. Participants were asked to circle, underline or cross out any words that stood out to them or that they would change. In addition, to facilitate active participation and discussion, participants were asked to rate each message on a scale of 1–5 (1 = not at all effective–5 = very effective), considering each of the following questions: “How effective would this message be in warning people about possible e-cigarette risks? In making people think twice about using e-cigarettes?” Participants then shared their ratings and statement perceptions with the group.

While the focus of discussion was on the proposed “hypothetical” warnings as described above, participants also later viewed an e-cigarette advertisement for one of two leading e-cigarette brands that already voluntarily include a warning statement in their advertising. Specifically, they viewed an ad from Vuse (2 groups), MarkTen (2 groups), or both (2 groups) and were asked about their perceptions of the ad and its included warning statement. The MarkTen ad featured a large image of a hand holding the e-cigarette, an image of the product and a text box on the bottom of the ad with four warning-related sentences preceded by the bolded word, “Warning”. The Vuse ad was a two page ad with a large image of the product, several product benefit claims in large font, and a two sentence small text warning located under the product image (which was not placed in a traditional warning text box). Images of the ads are available from the authors and from the Trinkets & Trash tobacco advertising collection [19,20].

After the session, participants were debriefed about the study, given a smoking cessation resource sheet, and a $50 gift card. This study was reviewed and approved by the Rutgers University Institutional Review Board.

### 2.4. Analysis

Sessions were transcribed and then coded using Atlas.ti qualitative software, using codes developed deductively a priori and inductively based on themes identified from repeated transcript readings. Two of the research team members (OW and a research assistant) coded two of the six transcripts together. The research assistant then coded the remaining four transcripts and OW reviewed these for agreement. Coded text was then re-reviewed to summarize the themes and patterns of participants’ responses and illustrative quotes were selected (in some cases edited for brevity and clarity).

## 3. Results

### 3.1. Warning Statement Ratings and Perceptions

#### 3.1.1. Message 1: Poisonous Liquid

Both current and non-e-cigarette users provided this warning with the highest effectiveness ratings, with 89% rating it a 4 or 5 (Table 1). The statement evoked strong reactions from participants across groups who described it as “scary” and “eye-opening”. At least one non-user joked that she would rather continue with her cigarettes if she saw this warning. The warning seemed particularly alarming to non-users not very familiar or experienced with e-cigarettes, as it raised new questions about the products for them. Several were confused and alarmed by the reference to a “liquid” in the product (“I didn’t like the word liquid… It just sticks out, like there is liquid in your cigarette?”; male, non-e-cigarette user, age 19). Participants were also alarmed by the “avoid contact with skin” messaging and some non-e-cigarette users appeared to misinterpret it, wondering if it meant the entire product could be dangerous to touch. Others (including e-cigarette users) considered that if something could be harmful to one’s skin, it likely would be harmful to one’s inside:

“If it can’t have contact with your outer skin, it shouldn’t have contact with your inner lungs. Usually they don’t have ’avoid contact with skin’ warnings unless it will like deteriorate something” (male, current e-cigarette user, age 23).

Although most current e-cigarette users gave the statement a high rating (recognizing it could scare people away), many noted that it might be somewhat misleading and exaggerated, since they personally had experience getting e-liquid on their skin or swallowing small quantities with no serious consequences (see Table 2, Part A). Some also did not think it was specific enough in terms of listing any actual health effects of e-cigarette use. While many participants across groups agreed that the warning could be effective for “everyone”, they also mentioned it would be important for people who had or were around children and for new e-cigarette users.

#### 3.1.2. Message 2: Contains Toxins

Most participants (61.1%) rated this warning as a 4 or 5, noting that it was truthful and concise. They explained that “toxins” referred to something that is dangerous, harmful, poisonous, or deadly, something that one should “stay away from”. Several thought the word “toxins” connoted something more harmful than “chemicals” (“I mean, a lot of things are chemicals, that doesn’t make them bad”; male, current e-cigarette user, age 19). However, a common critique was that the warning was not specific enough, that it should list actual toxins included and how they compare to those in cigarettes (in type and quantity). Some thought the word toxins was not strong enough and that “carcinogens” should be used instead.

Overall, several participants noted that this warning may be effective in warning new or potential e-cigarette users, including those who think e-cigarettes are relatively harmless. This could include new smokers and smokers looking to use e-cigarettes as a healthier alternative to smoking. However, one e-cigarette user pointed out that deterring smokers from switching to e-cigarettes could be a negative consequence of the warning:

“But that could also potentially stop people who are trying to switch…. then they keep smoking cigarettes which have significantly more toxins I’d be willing to bet.” (male, current e-cigarette user, age 19).

The more specific version of this statement received a higher average effectiveness rating (4.0) among participants in the two latter groups exposed to it (see Table 1), who found the references to lead and formaldehyde surprising and disturbing. Most agreed that this message might be more impactful with older audiences (who might be more familiar with the consequences of toxins such as lead and formaldehyde) than with young people who feel “invincible”.

#### 3.1.3. Message 3. Not Safe Alternative to Smoking

Over half of participants (59.3%) rated this statement as a 4 or 5. Many non-e-cigarette users (and a few current users) thought it could be a strong warning because people generally think e-cigarettes are safer than cigarettes, and that this statement countered those perceptions. As such, they thought it might warn and deter smokers interested in quitting cigarettes for health reasons from switching to e-cigarettes because the statement suggested that there would be no health benefit in doing so. Some e-cigarette users noted that even though they objectively believed the warning might deter others, it would not influence them personally:

“It wouldn’t change my mind, but for someone else it might because obviously it’s saying that it’s not a safer alternative to cigarettes, so then why go to it” (female, current e-cigarette user, age 35).

Alternatively, participants providing low ratings commented that it was not specific enough, i.e., it did not explain why it was not a safe alternative. Additionally, several e-cigarette users thought it would not be believable because people do think e-cigarettes are safer than tobacco cigarettes, either based on what they have heard or their own experiences. One e-cigarette user believed the statement was inaccurate and misleading:

“I think it’s a really intimidating warning label...You know, it’s not safe, it’s not completely harmless, but in comparison to cigarettes I definitely think it is safer. So this kind of I think gives the impression that it’s not safer than cigarettes.” (male, current e-cigarette user, age 19).

#### 3.1.4. Message 4: Contains Nicotine; Addictive

Participants were more mixed on their perceptions of the nicotine addiction warning label. High raters indicated that the statement was factual and straightforward, and would be important for individuals unaware that e-cigarettes contain nicotine and for warning smokers trying to quit that e-cigarettes were similarly addictive, a point that might deter some from wanting to use e-cigarettes (see Table 2, Part B). A few participants also noted that because some e-cigarettes do not contain nicotine, such a warning would help consumers know which product they were getting and its associated risks.

However, several participants across groups (including high raters) believed that the statement was not necessarily a strong one because “everyone already knows nicotine is addictive”, because smokers were addicted to nicotine anyway, and because it otherwise did not describe any serious consequences of use (see Table 2, Part C). A few participants also noted that the nicotine in e-cigarettes was part of their appeal, therefore pointing this out would not dissuade people from using them. Participants’ perceptions about nicotine being referred to as a “chemical” were also somewhat mixed. Some thought it stood out, was accurate, factual, and effective (“I think it’s a little scary”), while others thought it was not strong enough, suggesting words, such as “substance”, “carcinogen” and “killer”, instead. Although many participants did not believe this warning would impact current e-cigarette users, several nevertheless suggested that it could be important for non-smokers, new smokers, young people, and parents of young people.

#### 3.1.5. Message 5: Not Tested for Use in Quitting Smoking

Participants’ perceptions of this statement were also mixed. Many, particularly current e-cigarette users, believed it would not be meaningful or believable because e-cigarettes had personally helped them or others they know to quit smoking (see Table 2, Part D). Some also thought it should be up to the public (rather than some authority) to judge whether e-cigarettes work or not (“This statement means nothing to me just cause they haven’t tested it. I’ll test it myself,” female, non-user, age 58). Others thought the message simply was not a strong enough reason to dissuade them from using e-cigarettes, especially if they were not interested in e-cigarettes solely for quitting smoking. In contrast, others did find the statement somewhat alarming (“people need to know this”), although several seemed to interpret it as meaning that e-cigarettes had not been tested or approved for safety more generally (rather than for smoking cessation in particular) (see Table 2, Part E). It reminded them that they did not know what the ingredients of e-cigarettes are, nor what their potential effects might be.

#### 3.1.6. Message 6: Unknown Health Effects 

This statement received the lowest average rating (2.18). Several participants noted that it did not act as a warning because it did not provide any specific information or really “say anything”, and that it was generic and could be said of numerous products (“they could put that on a box of pizza”). Some seemed to question the credibility of the sender of such a statement (“This sounds like no one cares… why put a warning?”). One e-cigarette user noted that with such a statement, potential users might default to what they have heard themselves about e-cigarettes, which is generally that they are convenient, fun and safer than tobacco cigarettes.

Some participants agreed that editing the statement to refer to the “long-term” health effects of e-cigarettes as being unknown could strengthen it. Additionally, the two focus groups that viewed this alternatively worded statement rated it higher (3.89, see Table 1). Some participants referred to it as “accurate”, “honest”, and effective for cautioning potential users to proceed “at their own risk”.

### 3.2. Perceptions of Existing Warnings in E-Cigarette Ads

Participants who viewed the MarkTen ad largely began describing it by commenting on the warning label, which they found to be very noticeable and comprehensive. Participants agreed it could be an effective warning because it was specific about potential consequences and at-risk audiences (e.g., pregnant women) (see warning text in Table 3). However, while many appreciated its detail, they nevertheless agreed it might be too long to be read by audiences. However, some suggested that its length provided a heuristic that could visually communicate to a viewer that the product was dangerous, even if they did not read the full label. One participant commented that the label was alarming since it was longer than those appearing on cigarette packs/ads. In contrast, those who viewed the Vuse ad largely described it favorably, describing it as attractive, appealing and “cool”. For some, the nicotine addiction warning statement within the Vuse ad (see Table 3) did not stand out as being a warning label and did not detract from the ad. One person referred to it as the “fine print”, and several agreed that a general audience would likely not notice it. It was also not considered to be a strong warning for the same reasons given for the nicotine addiction warning tested earlier. Participants in groups that viewed both ads generally agreed that the Vuse ad (and product) was much more appealing.

Several people commented that these companies’ voluntary application of the depicted warnings were actions likely taken to try to “get ahead of the game” in anticipation of regulation that would require them to do so, and that the warnings offered the companies some protection from potential health related lawsuits from users. Several also suggested that the move could make the companies look like they have nothing to hide, like they “care” about consumers and ultimately be more trustworthy.

“It makes you wanna trust them more…over a different company. They’re telling you what’s in the product, instead of other companies making you guess and research yourself…” (male, current e-cigarette user, age 23).

## 4. Discussion

In its recently published deeming rule extending its authority to e-cigarettes, the FDA only required one nicotine addiction warning to be applied to e-cigarettes (a simplified version of the one tested in this study) [6]. However, the deeming rule notes that the nicotine addiction warning is a “minimum required warning statement” and that the rule does not preempt states nor preclude e-cigarette manufacturers from additionally retaining or adding their own warnings that are truthful and not misleading. It also acknowledges that the use of a single health warning could allow it to grow stale over time and indicates that the FDA “intends to conduct research and keep abreast of scientific developments” regarding the warning’s efficacy, information it could use to revise the warning or add any additional warning statements in a future rulemaking [6]. To our knowledge, this is the first qualitative study of warning statements about e-cigarettes, and data from this study can be used to help understand how consumers might interpret various e-cigarette warnings and to inform future work on this topic.

We found that both current smokers and e-cigarette users appeared generally open to the idea of e-cigarette warning labels and provided constructive feedback on concept statements. In fact, a major theme of our findings was that the presented statements were not perceived as being specific enough about the risks of e-cigarettes. Participants wanted references to actual ingredients and toxins included (and levels of these) and to e-cigarette health effects. This is consistent with recommendations that tobacco warnings be about specific risks over general references to harm [3,4]. However, this will be a challenge for e-cigarettes given that the full range of health effects, particularly long-term effects, are not yet scientifically known, and that the types and levels of toxins present in e-cigarettes can vary greatly by e-cigarette type [21]. Future research should continue to investigate whether certain more general warnings or warnings that express some level of uncertainty may be appropriate while still being more effective than no warnings at all. One of the previous experimental studies found that the presence of any e-cigarette warning reduced young adults’ intentions to purchase an e-cigarette over none at all [13].

On the other hand, we also found that statements that were perceived as “stronger” and “scarier” were seen by some participants as messages that could potentially deter smokers from switching to less harmful e-cigarettes, which could have a negative public health effect if those people otherwise continue to smoke. One of the statements examined in this study (“this product is not a safe alternative to cigarettes”), which is the same warning message currently used for smokeless tobacco products and to be extended to cigar products, was misinterpreted by many as suggesting that e-cigarettes are not safer than cigarettes and should likely be avoided in future e-cigarette warning proposals or educational campaigns given that it may be potentially misleading.

Results from this study suggest that in any future development of additional warnings for e-cigarettes, it will be important for the tobacco control community to find an appropriate balance given the unique factors associated with e-cigarettes. While the main behavioral goals of cigarette warning labels are to encourage smokers to quit and prevent non-smokers from starting, a parallel set of goals for e-cigarettes (i.e., encourage all current e-cigarette users to quit and prevent non-users from starting) may not be appropriate given their potential use for smoking harm reduction. Overall, it would seem that a common goal of e-cigarette warnings for all audience types could be to increase peoples’ awareness/knowledge about their potential for addiction and increase understanding that they are not necessarily risk-free. However, if e-cigarettes can play a meaningful role in harm reduction, then ideally, such warnings should also discourage e-cigarette interest and use among never smokers while not discouraging use among current and former smokers interested in e-cigarettes for help in quitting smoking or maintaining smoking abstinence. In our study, we found that many of the current e-cigarette users acknowledged that the various warning messages might be important, particularly for some audiences, but that it would not personally make them stop using e-cigarettes. This would appear to be consistent with the “ideal” warning effect mentioned above, although quitting smoking/reducing harm was not these users’ only reasons for using e-cigarettes. The potential impact of these statements on the risk perceptions and use intentions of different audience types should be explored with larger samples and with experimental designs.

Regardless of what e-cigarette warnings may be used, display considerations are also important. Based on reactions to some current e-cigarette ads, our results also suggest that at a minimum, e-cigarette warnings should be required to be clearly labeled in visible text boxes with bolded black borders (similar to those used for cigarette and smokeless tobacco products) so that they may be more noticeable and recognized as warnings. This is consistent with good label practices, as the use of such boxes has been found to increase the salience and recall of warnings [1] and is also consistent with the warning requirements for newly deemed tobacco products (including e-cigarettes) published in the recent FDA deeming rule [6]. It was also suggested during our study that a good e-cigarette warning might be a combination of the various statements presented. While this might make up for some of the lack in specificity of the individual messages and serve as a visual heuristic communicating product risk, there are also implications for warning statement length related to readability and label placement that might not be practical. In addition, if using e-cigarette warnings that are longer than those for other tobacco products leads to perceptions that they are equally or more harmful than cigarettes, this might not be appropriate.

This study had several limitations including use of a small convenience sample of New Jersey residents, which limits the generalizability of the results. In addition, our sample was limited to current smokers and current e-cigarette users (including dual product users), and did not include non-smokers nor youth, who, as noted above, are also critically important audiences for e-cigarette warnings. The hypothetical warning statements were viewed by participants in a laboratory setting (versus in a real world setting) and were viewed in isolation (not on packs or advertisements), methods which limit the finding’s external validity. Additionally, perceived effectiveness ratings are commonly used in formative media message work [5,22,23] and our use of a “perceived effectiveness” rating activity was intended to help stimulate participants’ active participation and group discussion and to generate ideas for future research exploration rather than to provide any precise quantitative results about the relative effectiveness of the different messages examined. And while resulting discussions from this activity provided insight into what the impact of such messages might be on e-cigarette risk perceptions and use intentions, future studies should more formally evaluate the potential impact of e-cigarette warnings on attitudes and beliefs, use intentions and other relevant constructs (e.g., attention and recall).

## 5. Conclusions

This study provides the first qualitative insights about potential reactions to different types of e-cigarette warning statements, and important insights into unique challenges and considerations for their development. In addition, although our study design explored participants’ perceptions about the statements as potential future e-cigarette warning labels, our results may also provide some insight into how smokers and e-cigarette users may perceive such statements when and if they are encountered in other contexts (e.g., through other media or interpersonal channels). Given that scientific knowledge about any potential health effects of e-cigarettes is still evolving, future research should continue to explore whether certain general warnings about potential risks (e.g., may include toxins) or about risk uncertainty (e.g., unknown health effects) may be useful and appropriate in the FDA’s goal to help consumers “understand and appreciate” the risks of using tobacco products through warnings [6]. Development of future warnings should also take care to balance the need to inform about potential risks with their potential to serve as harm reduction products. Meanwhile, the FDA could consider rotating multiple differently worded nicotine addiction warnings (e.g., emphasizing effects on adolescent brain development, written in testimonial style) to better reach different audience types and avoid message wear out [1]. Future research should continue to explore reactions to and potential effects of these and additional e-cigarette warning statements with various audience types.

## Figures and Tables

**Table 1:** Current e-cigarette users’ and non-e-cigarette users’ ratings of proposed e-cigarette warning statements on a scale of 1–5 (1 = not at all effective, 5 = very effective).

Presented Warning Statements		% Rated as 1 or 2	% Rated as 3	% Rated as 4 or 5	Average Rating
Message 1 Warning: The liquid in this product includes chemicals. Poisonous if swallowed. Avoid contact with skin	**Total**	**3.7**	**7.4**	**88.9**	**4.44**
	Non-users	0	7.7	92.3	4.77
	Current users	7.1	7.1	85.7	4.14
Message 2 Warning: E-cigarette vapor contains known toxins	**Total**	**16.7**	**22.2**	**61.1**	**3.55**
	Non-users	20	10	70	3.7
	Current users	12.5	37.5	50	3.375
Message 2a (Alternative version of above ^1^): Warning: E-cigarettes contain at least 10 toxic substances including lead and formaldehyde ^2^	**Total**	**22.2**	**11.1**	**66.7**	**4**
	Non-users	0	0	100	5
	Current users	33.3	16.7	50	3.5
Message 3 Warning: This product is not a safe alternative to cigarettes ^3^	**Total**	**22.2**	**18.5**	**59.3**	**3.44**
	Non-users	15.4	23	61.5	3.8
	Current users	28.5	14.3	57.1	3.14
Message 4 Warning: This product contains nicotine derived from tobacco. Nicotine is an addictive chemical ^4^	**Total**	**44**	**12**	**44**	**3.16**
	Non-users	30.8	15.4	53.8	3.38
	Current users	58.3	8.3	33.3	2.92
Message 5 Warning: This product has not been tested or approved for use in quitting smoking ^5^	**Total**	**44.4**	**22.2**	**33.3**	**2.7**
	Non-users	38.5	38.5	23	2.61
	Current users	50.00	7.1	42.9	2.79
Message 6 Warning: The health effects of e-cigarettes are unknown	**Total**	**64.7**	**17.6**	**17.6**	**2.18**
	Non-users	55.5	33.3	11.1	2.22
	Current users	75	0	25	2.125
Message 6a (Alternative version of above ^1^) Warning: The *long-term* health effects of e-cigarettes are unknown	**Total**	**11.1**	**33.3**	**55.5**	**3.89**
	Non-users	0	66.7	33.3	3.67
	Current users	16.7	16.7	66.6	4

^1^ Alternatively worded version of warning statement viewed by two of six focus groups; ^2^ Statement tested in the Sanders-Jackson et al. study (2015) [13]; ^3^ Statement tested in the Popova and Ling study (2015) [14]; ^4^ Warning originally proposed for e-cigarettes by FDA in its April 2014 proposed deeming rule; ^5^ Similar to warning used in Canada: “Health Canada has not approved this product for quitting smoking”.

**Table 2:** Select example quotes of participants’ message perceptions, by message and theme.

**A. Perceptions that Message 1 might be exaggerated:** “It spilled on my hands before and nothing ever happened, and I also like, it leaked in my mouth and I’m still here”. (female, current e-cigarette user, age 25) “I mean that’s kind of just a good way to deter people from buying the product, but I think that’s maybe too much of a warning…that immediately puts the thought in your head like, ‘if I spill this on myself, is my finger gonna dissolve?’ That’s more just scary”. (male, current e-cigarette user, age 20)
**B. Perceptions that Message 4 might dissuade smokers:** “I gave it a 5 because I think most people are under the assumption, as I, that they don’t contain nicotine. So therefore, if you’re telling me that it *does* contain nicotine, I think it’s going to be effective. It will veer people away that want to quit smoking, you know?” (female, non-e-cigarette user, age 57) “My juice doesn’t say this, but I think if it was on it, some people would be like ‘oh, this is gonna be addictive? I don’t want this cause you know I’m trying to stop smoking cigarettes’. (female, current e-cigarette user, age 26)
**C. Perceptions that Message 4 is not strong enough:** “It is not striking me enough to not touch that. Ok nicotine, I see everybody is smoking them so it can’t be that bad. So that is why it is not effective”. (female, non-e-cigarette user, age 50) “Everyone knows nicotine is addictive. This label means nothing to me. It doesn’t say something like you know, “you’ll lose your teeth” or something like that...”.(male, current e-cigarette user, age 23)
**D. Perceptions that Message 5 may not be effective because e-cigarettes do help people quit** “You know people that know somebody that is vaping now or using an e-cig that hasn’t picked up a cigarette in a long time… you’ve seen it tested and you’ve seen it work…”(male, current e-cigarette user, age 26) “I gave it a 1…I’m not buying it, because it did help me quit smoking, so I would say this is b.s.”. (female, current e-cigarette user, age 33)
**E. Perceptions that Message 5 may suggest e-cigarettes haven’t been tested/approved for safety more generally** “I gave it a 4 too because you are telling me this has not been tested at all, I am kinda scared about that…most people go, ‘What? It hasn’t been tested?’ So I don’t know what I am getting myself into, what effects it may take on me”. (female, non-e-cigarette user, age 50) “Um the words ‘tested’ and ‘approved’ stood out to me and like kinda just gives off like that they didn’t test it at all, so it’s just like experimental. But uh I mean every vape I’ve ever smoked, I kinda never looked to see if it had a label or if it’s been tested or approved. I mean I don’t really know anybody that’s like actually researched if it’s been approved for use in quitting smoking or it’s been like approved in any way”. (male, current e-cigarette user, age 20)

**Table 3:** Voluntary warning messages in presented Vuse and MarkTen e-cigarette print ads.

Brand	Warning Message
Vuse	“Vuse contains nicotine extracted from the tobacco plant. Nicotine is addictive and no tobacco product has been shown to be safe.”
MarkTen	“Warning: This product is not intended for use by women who are pregnant or breast feeding, or persons with or at risk of heart disease, high blood pressure, diabetes, or taking medicine for depression or asthma. Nicotine is addictive and habit forming, and it is very toxic by inhalation. Nicotine can increase your heart rate and blood pressure and cause dizziness, nausea, and stomach pain. Inhalation of this product may aggravate existing respiratory conditions.”
